# Analysing Microbial Community Composition through Amplicon Sequencing: From Sampling to Hypothesis Testing

**DOI:** 10.3389/fmicb.2017.01561

**Published:** 2017-09-04

**Authors:** Luisa W. Hugerth, Anders F. Andersson

**Affiliations:** ^1^Department of Molecular, Tumour and Cell Biology, Centre for Translational Microbiome Research, Karolinska Institutet Solna, Sweden; ^2^Division of Gene Technology, Science for Life Laboratory, School of Biotechnology, KTH Royal Institute of Technology Solna, Sweden

**Keywords:** bioinformatics, biostatistics, amplicon sequencing, microbiome, NGS, 16S rRNA, microbial ecology

## Abstract

Microbial ecology as a scientific field is fundamentally driven by technological advance. The past decade's revolution in DNA sequencing cost and throughput has made it possible for most research groups to map microbial community composition in environments of interest. However, the computational and statistical methodology required to analyse this kind of data is often not part of the biologist training. In this review, we give a historical perspective on the use of sequencing data in microbial ecology and restate the current need for this method; but also highlight the major caveats with standard practices for handling these data, from sample collection and library preparation to statistical analysis. Further, we outline the main new analytical tools that have been developed in the past few years to bypass these caveats, as well as highlight the major requirements of common statistical practices and the extent to which they are applicable to microbial data. Besides delving into the meaning of select alpha- and beta-diversity measures, we give special consideration to techniques for finding the main drivers of community dissimilarity and for interaction network construction. While every project design has specific needs, this review should serve as a starting point for considering what options are available.

## A brief history of methodologies for determining microbial community composition

While humans have been selectively breeding bacteria and fungi for food fermentation for several centuries, the first observations of microbial organisms were made in the 1670's by Antony van Leeuwenhoek, who first observed microbes (that he called “animalcules”) in saliva, and the first purposeful and successful isolation of bacteria for scientific purposes was attained by Robert Koch and Julius Petri in the 1870's. Both direct observation and culturing remain invaluable techniques to this day, albeit both have limitations.

Culturing is the gold standard for microbial characterization, as it provides large amounts of cells from a clonal population, and allows any number of functional tests on bacterial biochemistry, physiology and genetics to be performed. It was however evident even to Koch that different bacteria grow best in different settings, and by the early 1900's it was accepted that the vast majority of bacteria could not be cultivated with standard techniques, a phenomenon later dubbed “the great plate count anomaly” (Staley and Konopka, 1985). Therefore, most of what is known today about bacterial physiology stems from a very small subset of easily culturable bacteria of medical or veterinary importance which grow well in the presence of high nutrient loads (Lagier et al., 2015).

Reasons for refraction to culturing are many. Firstly, in the absence of knowledge of the specific growth requirements of an organism, trial-and-error is not a feasible way to determine them (Stewart, 2012), especially considering that many organisms have rather narrow windows of growth (Lagier et al., 2015). These microbes might survive in the environment in boom-and-bust cycles (Iluz et al., 2009; Gilbert et al., 2012) or grow at a pace so slow as to be nearly indistinguishable in the lab (Zengler et al., 2002; Lagier et al., 2015).

Laboratory settings can also generate toxic conditions, such as oxidative stress (Morris et al., 2011; Tanaka et al., 2014). In addition to this, organisms might fail to grow due to missing pathways (Nye et al., 1999), or be dependent on siderophores produced by other members of their community (D'Onofrio et al., 2010).

Even today, despite high-throughput dilution-to-extinction culturing techniques (Aakra et al., 1999; Rappé et al., 2002; Aoi et al., 2009; Liu et al., 2009), culture chambers that mimic natural environments (Zengler et al., 2002; Nichols et al., 2010; Sizova et al., 2012) and co-culturing approaches (Kaeberlein et al., 2002; Tanaka et al., 2004; Morris et al., 2008), isolating and culturing bacteria is a complex and time-consuming endeavor.

An alternative to culturing is to perform microscopy directly on environmental samples. High-resolution microscopy techniques such as electron microscopy, confocal microscopy and photoswitchable fluorophores allow a number of specific biological questions to be addressed directly from images of live or fixated bacteria (reviewed in Coltharp and Xiao, 2012). However, regardless of technology, with observation alone it can be extremely hard to achieve reasonable functional or taxonomic resolution for the diversity of microbes typically found in an environmental sample. It takes years of training as a taxonomist to excel in the visual identification of microbes, even ones with as much morphological diversity as protists; and even then there are strong observer effects (reviewed in Moreira and López-García, 2002; Silva, 2008).

To move beyond the difficulties of culturing and the limitations of microscopy, microbial ecologists have moved increasingly toward molecular fingerprinting. Starting in 1977, Woese and colleagues established the suitability of the small subunit (SSU) of the ribosomal RNA (rRNA) gene for inferring phylogenetic relationships between prokaryotic organisms, a property later verified to also apply to eukaryotes (Woese and Fox, 1977; Woese et al., 1985; Woese, 1987). Norman Pace and colleagues soon started applying the same technique to natural communities (Pace et al., 1985; Stahl et al., 1985). Together with the ribosomal internal transcribed spacer (ITS), this is still the most commonly used gene for community phylogenetic composition analysis (community fingerprinting). The advantages of using SSU rRNA for community fingerprinting are many: (i) This gene is found in all cellular life forms. (ii) It is a highly conserved gene, serving to a large degree as a reliable molecular chronometer. (iii) It is seldom transferred horizontally. (iv) It possesses both conserved and variable regions, so that the conserved regions can be targeted by polymerase chain reaction (PCR) primers and the variable ones be used as identifying markers. A handful of other genes, such as the large subunit (LSU) rRNA share these properties, but the length of ~1,500 bp of the bacterial SSU rRNA made it amenable to early molecular techniques, and the impressive body of knowledge that has since accumulated on the basis of this gene makes a switch to other markers very impractical, except in certain sub-fields such as mycology, where ITS and LSU are widely used.

The 1990s saw the first high-throughput environmental fingerprinting approaches, sometimes referred to as microbiomics. It is the decade of techniques such as denaturing gradient gel electrophoresis (DGGE) (Muyzer et al., 1993), terminal restriction fragment length polymorphism (T-RFLP) (Liu et al., 1997) and automated ribosomal intergenic space analysis (ARISA) (Fisher and Triplett, 1999), all of which are based on the characteristic travel distance of PCR amplified DNA fragments (amplicons) in an electrophoretic device. These banding patterns can be used directly to compare broad changes in taxonomic composition of samples in different conditions. Even though, in each of these techniques, different organisms might give rise to identical bands, each band is treated as an operational taxonomic unit (OTU). To assign a tentative taxonomy to the OTU, high abundance bands can be selected for sequencing.

At around the same time, microarrays emerged as an alternative to fingerprinting. A downside of microarrays is that identification is restricted to sequences previously known and printed onto the array (Ehrenreich, 2006). While this limits its applications as a general environmental survey tool, microarrays can still be valuable tools in focused clinical, industrial or environmental monitoring settings (Humbert et al., 2010; Ricke et al., 2013; Zumla et al., 2014). Since microarrays can cover various regions of the genome, they can be used for distinguishing between closely related species or strains (Lehner et al., 2005; Singh and Mohapatra, 2008; Narihiro and Sekiguchi, 2011).

The rise of high-throughput DNA sequencing was a game changer for microbial ecology. In 2006, the first study was published using 454 pyrosequencing for assessing microbial communities, a survey of the microbial diversity in a marine water community (Sogin et al., 2006). This study, while sequencing relatively shallowly (6,505–22,994 sequences/sample), already presented two of the main characteristics of sequencing-based microbiomics that came to be seen as standards in the field: rarefaction curves very far from reaching saturation, which indicated a much larger microbial diversity than previously suspected; and a highly uneven community, with 3–4 orders of magnitude of difference in abundance between the least and most abundant tags. These previously unknown low abundance organisms were dubbed in the paper the “rare biosphere”, a term still in use and whose biological relevance is much discussed (Lynch and Neufeld, 2015). Later, the introduction of sample-specific barcode sequences (Andersson et al., 2008; Hamady et al., 2008) allowed sequencing many samples in the same run and opened up the door to large-scale comparative microbiome studies.

## Sample collection, storage, DNA extraction, library preparation and sequencing

While high-throughput amplicon-sequencing has proven powerful and accurate for microbial community analysis, random and systematic errors can be introduced in several of the steps along the analysis chain. Sampling itself can introduce biases, which has to be considered when collecting or analysing any sort of ecological data. Solid samples such as soil can have extreme short-distance heterogeneity (Certini et al., 2004). The amount of material used for extraction, and the definition of the sample (e.g., whether they're homogenized in bulk or kept separately) has to be suited to the research question at hand. As for aquatic samples, long term studies must contend with the issue of the flowing and mixing of water masses. A stationary sampling, fixed to geographical coordinates, faces the issue that changes observed in the microbial community can be a true change within a community or a replacement of one community by another as the water flows. As an alternative to the stationary eulerian sampling, it is possible to follow a water mass using a buoy and collect samples around it, a strategy termed Lagrangian sampling. This approach, however, is only effective for a few weeks, after which the water mass is mixed beyond the point where it can be considered coherent with the initial sample. The temporal dimension is crucial regardless of the sampling strategy, since the frequency of sampling should be (but often isn't) commensurate with the rate of the biological processes of interest.

Sample storage is also extremely important, to prevent bacterial overgrowth as well as taxonomically biased DNA damage or degradation. Bacteria should be inactivated as soon as possible without causing significant damage to their DNA (Choo et al., 2015; Song et al., 2016). While most laboratories correctly choose to keep their samples in the freezer, it is also necessary to consider the damage done by repeatedly freezing and thawing cells (Moré et al., 1994; Harju et al., 2004) and DNA (Thomson et al., 2010; Todorova et al., 2012). While short amplicons can usually still be amplified even from fragmented DNA, long amplicons, metagenomic libraries, and cDNA libraries have stricter requirements. Researchers should assess their study design and laboratory capacity and consider the possibility of storing samples in an appropriate preservation medium (Roberts, 2016).

**Figure 1:**
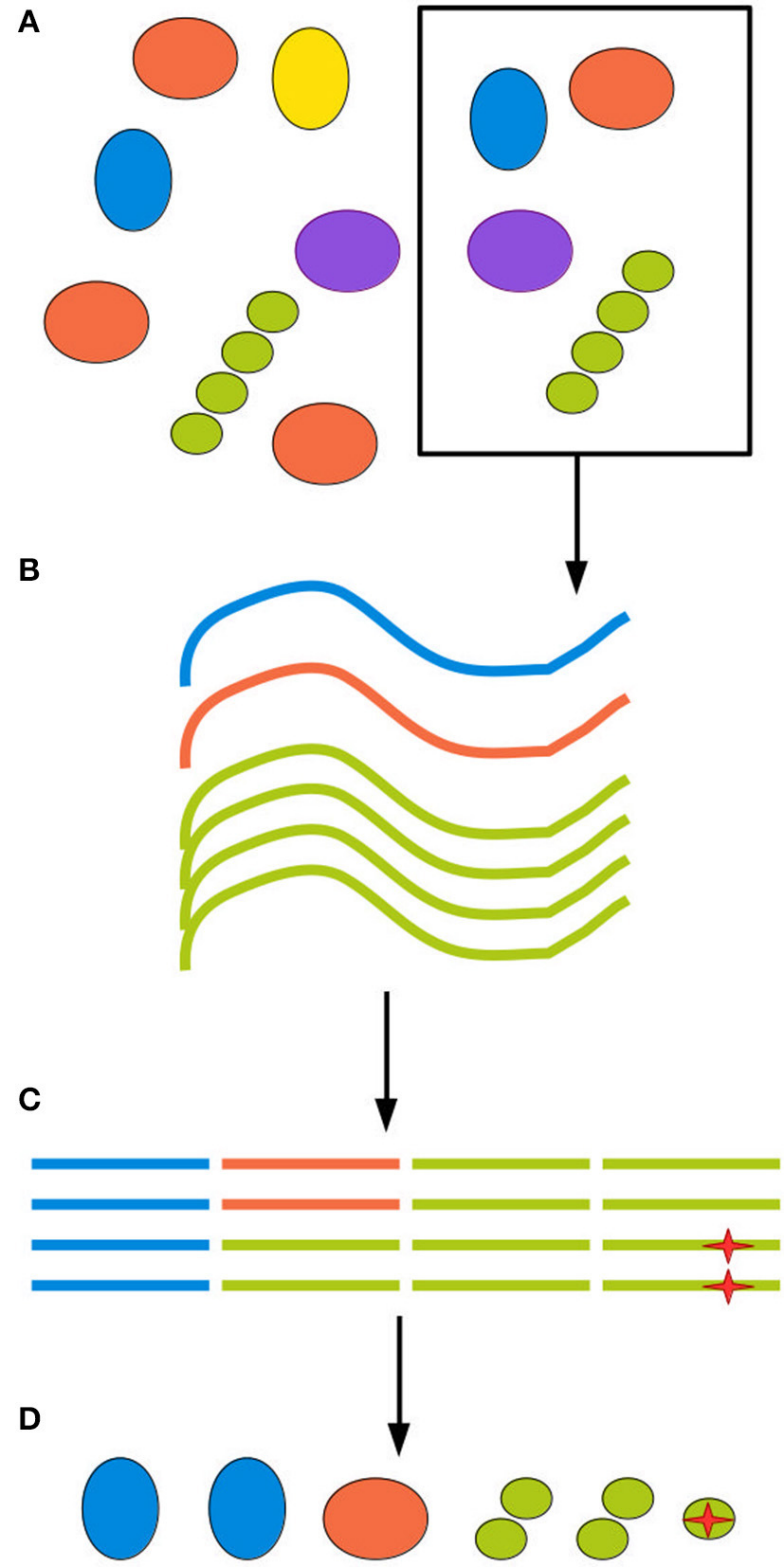
All experimental steps can contribute to the total bias. **(A)** When retrieving an environmental sample, not all clades present in the environment will be present in the sample, and those who are present might be present in different proportions than in the environment as a whole. **(B)** DNA extraction methods inevitably have different efficiency rates for different clades, further distorting the sample. **(C)** Finally, amplification and sequencing both introduce biases and point mutations, represented here by a red star, as well as possible chimerism. **(D)** The final inferred community can be quite different from the ground truth.

The next source of bias and artifacts is the DNA extraction method. Extraction relates to sampling, since different methods require and tolerate different amounts of starting material. The physico-chemical characteristics of the environment and of the biological material in it will in turn interact with the extraction method, producing a more or less efficient disruption of cell walls and membranes and removal of contaminants. A failure to appropriately disrupt certain types of cell wall will cause those organisms to be underestimated in the community profile. A failure to remove contaminants such as other biomolecules and organic acids will inhibit the DNA amplification step, leading to amplification biases and eventually even sample loss (Weiss et al., 2014; Gorzelak et al., 2015; Reck et al., 2015; Walker et al., 2015). Finally, for samples of low microbial density, such as patient blood samples, minute amounts of DNA or cellular contamination in any reagent or piece of equipment used in extraction will generate spurious reads (Salter et al., 2014). Figure 1 illustrates biases at each step of the sampling procedure.

It must also be noted that RNA can also be extracted and analyzed as complementary DNA (cDNA). DNA is a more stable molecule, so community signatures are less likely to experience radical change at the DNA level as a result of sample collection (Lim et al., 2014; Reck et al., 2015). On the other hand, different organisms, specially eukaryotes, can have an enormous range of copies of the rRNA gene in their genomes, which hinders a simple correlation between gene copies and cell numbers (Gong et al., 2013). The number of rRNA copies per cell, however, is largely independent of the number of gene copies in prokaryotic cells and is instead correlated to cell activity (Jones and Lennon, 2010). Activity, as measured by the ratio of 16S rRNA:16S rRNA gene copies, has in turn been shown to often be highest in low-abundance populations within a community (Campbell et al., 2011; Zhang et al., 2014). These differences can result in very different community profiles for cDNA and DNA analyses, for example in deep water layers, which at the DNA level are more affected by sinking dead and otherwise inactive cells (Zhang et al., 2014; Cram et al., 2015). When comparing cDNA and DNA profiles from spatially heterogenous biomes (such as soil), it may be important to do both types of analysis on the same actual samples (Roume et al., 2013).

After nucleic acid extraction (and cDNA synthesis when applicable), the region of interest must be amplified and prepared for sequencing. This is almost always achieved through PCR, a method which is sturdy and cost-effective, but may introduce large biases to the sample (Schirmer et al., 2015). PCR depends on primers, short DNA molecules (usually 15–30 bp) of defined sequence that bind to the ends of the DNA target region on the template strands and allow a DNA polymerase to synthesize a new DNA strand, complementary to the template and downstream of the primer's 3′ end. By flanking the region of interest with two primers, its copy number is doubled at every polymerization cycle, hence the term “polymerase chain reaction”. This means that a DNA template which does not present complementarity to the primers will not be amplified and its corresponding organism will be a false negative in the microbiome profile. By applying a mixture of primers with base-level variations (degenerate primers), the percentage of taxa being targeted by the primers can be increased. On the other hand, this increases the odds of amplifying other DNA regions, creating artificial diversity. Furthermore, although mismatches in the primer sequence are the main cause of amplification bias, preferential binding of sequences containing C/G rather than A/T at a degenerate position is also a strong factor (Lanzén et al., 2011). Therefore, the exact sequence of the PCR primer should be considered in terms of the community at hand and the acceptability of different biases in the resulting amplicon pool. The choice of primer is also very sensitive since the same community amplified with different high-quality primer pairs will still give a different profile, since different lineages may evolve at different rates in each variable region of their marker gene. Thus, studies focusing on different subregions aren't directly comparable (Nossa et al., 2010; Soergel et al., 2012; Yang et al., 2016) and some studies even choose to analyse different variable regions in parallel (Smith et al., 2012).

Several papers have been published that systematically assess the ability of primer sequences to amplify 16S (Baker et al., 2003; Wang and Qian, 2009; Youssef et al., 2009; Gantner et al., 2010; Nossa et al., 2010; Kumar et al., 2011; Soergel et al., 2012; Klindworth et al., 2013), 18S (Amaral-Zettler et al., 2009; Stoeck et al., 2010; Hugerth et al., 2014a), fungal ITS (Martin and Rygiewicz, 2005; Manter and Vivanco, 2007; Toju et al., 2012; Op De Beeck et al., 2014) and many other genes. PrimerProspector (Walters et al., 2011) and DegePrime (Hugerth et al., 2014b) are examples of computer programs that can aid in the design of broad taxonomic primers. DegePrime finds the primers with maximal taxonomic coverage while controlling the degeneracy to a user-specified level.

Another major issue with PCR is the formation of chimeras, that is, DNA molecules containing partial sequences from two or more biological sequences. This arises due to sequence similarity between amplicons and the nature of the chain reaction. Partially amplified molecules might break and anneal unspecifically to templates from other lineages, serving as primers for new, chimeric sequences. Factors that have been linked to increased frequency of chimeras include both laboratory settings, such as fast thermocycling during PCR (Stevens et al., 2013), and intrinsic characteristics of the sample, such as richness and diversity (Fonseca et al., 2012).

As more and more research groups started using short-read high-throughput gene tag sequencing, first with 454 pyrosequencing and later with Illumina and Ion Torrent technologies, it also became increasingly clear that these methods, while less biased than some of their predecessors, do produce a considerable number of artifacts, which can be very hard to detect and separate from true biological signal. As an example of this, by using the same filtering strategy as the seminal work of Sogin et al. (2006), Quince et al. (2011) observed c. three times more 97%-clustered OTU than were present in the mock community used. Three main kinds of errors can arise from PCR amplification and sequencing: substitutions (a base is read in place of another), insertions (a base is read more times than were actually present) and deletions (a base is skipped). Each sequencing platform has its characteristic error profiles and assorted suite of tools for handling them, which have been described elsewhere (Quince et al., 2009; Gilles et al., 2011; Carneiro et al., 2012; Bragg et al., 2013; Laver et al., 2015; Schirmer et al., 2015). In addition to this, reads from one sample might be assigned to a different one, due to contamination or sample-switching (exchange of index during library preparation or sequencing). This issue has been estimated to affect up to 2% of reads in certain datasets, and is hard to control for (Edgar, 2016a).

In addition to sampling and library preparation, the choice of sequencing platform has to be suitable for the environment and research questions of interest. Longer reads can increase the accuracy of phylogenetic placement (Okubo et al., 2012; Quick et al., 2015), while a larger number of reads might be needed to reduce the effect of random noise and increases the sensitivity of the approach. In addition to the short read technologies which are the focus of this work, long read approaches such as PacBio (Schloss et al., 2016) and Oxford Nanopore MinION are increasingly in use (Benítez-Páez et al., 2016; Lindberg et al., 2016; Hu et al., 2017).

## OTU clustering and taxonomic annotation

The initial sequencing data processing step is filtering based on read quality scores and the presence of the expected primer and adapter sequences, and the removal of these non-biological sequences. Commonly used tools for this task are Fastx (Gordon and Hannon, 2009), TrimGalore (Krueger, 2017) and Cutadapt (Martin, 2012). Low quality bases, adapters, primer dimers, reads that are too short and obvious contaminants (e.g., human DNA) need to be removed. Then, for paired-end reads, it is common to merge them at their overlapping regions. Since read quality also falls toward the end of the read for most short-read technologies (Salipante et al., 2014; Schirmer et al., 2015), this is also a way to increase the confidence in the bases in this region (Salipante et al., 2014). Good stand-alone tools for this are Usearch (Edgar, 2013) and FLASH (Magoč and Salzberg, 2011), and it can also be achieved using MOTHUR or Qiime (Schloss et al., 2009; Caporaso et al., 2010). Single-end short reads should be trimmed to the same length for comparability, and reads shorter than the cutoff, discarded (Edgar, 2013). This approach can also be used for paired-end reads covering a region too long for merging; in this case, they can be trimmed to a fixed length and concatenated (Hugerth et al., 2014a). FastQC (Andrews, 2009) or MultiQC (Ewels et al., 2016) can be used to assess the quality of the data before and after quality filtering, including whether errors are randomly distributed or clustered at certain bases or regions of the flow cell.

The next step is usually to “pick OTU”. In the case of sequencing, OTU are most often defined by clustering sequences according to similarity. This step is meant to eliminate erroneous sequences formed by PCR and sequencing errors, since these should deviate from a true sequence by only a few bases. This way, a sequence diversity is reduced to true biological diversity. Since small variations in sequence are observed among strains of a single species, and even among different operons of a single strain, it is assumed that tags differing by only a small percentage of their bases represent taxonomically equivalent cells. This is not always true, however, as in the well documented case of Escherichia *spp. a*nd S*higella spp.*, which despite having clearly distinct natural histories harbor the exact same sequence along the full length of their 16S rRNA gene (Zuo et al., 2013).

OTU picking procedures can be divided into closed reference, open reference and *de novo*. Closed reference means mapping reads to a database and assigning them to the best possible match. Reads that do not match with a sufficiently high score are discarded. On an open reference approach, those sequences that fail to match to the reference are submitted to a *de novo a*pproach.

*De novo* approaches, in their turn, can broadly be divided into hierarchical clustering (based on single, average or complete linkage) (Schloss and Handelsman, 2005) and heuristic strategies. In single-linkage, a sequence is placed in a cluster if it has a similarity above a threshold to at least one other sequence in the cluster. This procedure tends to form very large clusters with a lot of heterogeneity and is rarely used, except occasionally for very rapidly evolving genes such as the fungal ITS region (Lindahl et al., 2013). Complete linkage, on the other hand, requires that a sequence in a cluster has similarity above the threshold to all others. This method produces therefore much more and smaller OTU than the other, and tends to overestimate measures of community richness, especially for data with many errors. For average-linkage, finally, the average similarity between a sequence and all others in the same cluster has to be above the threshold.

Since it is computationally very demanding to run an all-against-all comparison on datasets of millions of reads, as is done in hierarchical clustering, heuristic approaches were developed, the most relevant of which being the Usearch/Uparse suite (Edgar, 2010). It approximates complete- or average-linkage approaches by only comparing each sequence to a “centroid” sequence within each cluster. By selecting a distance cutoff between this centroid sequence and the candidate sequences, an average-linkage clustering is approximated. A recent benchmarking found that average-linkage clustering produced the most meaningful and stable OTU, followed by the distance-based greedy clustering implemented in Usearch (Westcott and Schloss, 2015). A consequence of this is that the order in which sequences are handled affects the final result. Therefore, sequences are generally sorted by decreasing abundance before clustering, since abundant sequences are less likely to be artifacts. From the description of these methods, it is clear that the distribution of distances between sequences in clusters will differ depending on the approach used, although the same nominal similarity cutoff is applied, a fact that is often glossed over when discussing microbiomics.

Hierarchical clustering was first made widely available to the ecology community through the software DOTUR (Schloss and Handelsman, 2005), but can now be found in many implementations, most prominently its successor MOTHUR (Schloss et al., 2009) and the CD-HIT package (Fu et al., 2012). As for heuristic strategies, popular software packages for OTU picking are Usearch (Edgar, 2013), Qiime (Caporaso et al., 2010), which runs Uclust in the background, and Vsearch (Westcott and Schloss, 2015), an open-source alternative to Usearch. However, these and other approaches suffer from OTU instability, that is, the fact that the same sequence might be assigned to different OTU depending on the community context (He et al., 2015; Schmidt et al., 2015). Most OTU-picking pipelines include a chimera removal step, either by comparing sequences to a known database, or d*e novo* by flagging low abundance sequences that could be formed by a combination of two sequences of higher abundance within the dataset (Schloss et al., 2009; Caporaso et al., 2010; Edgar, 2010).

**Figure 2:**
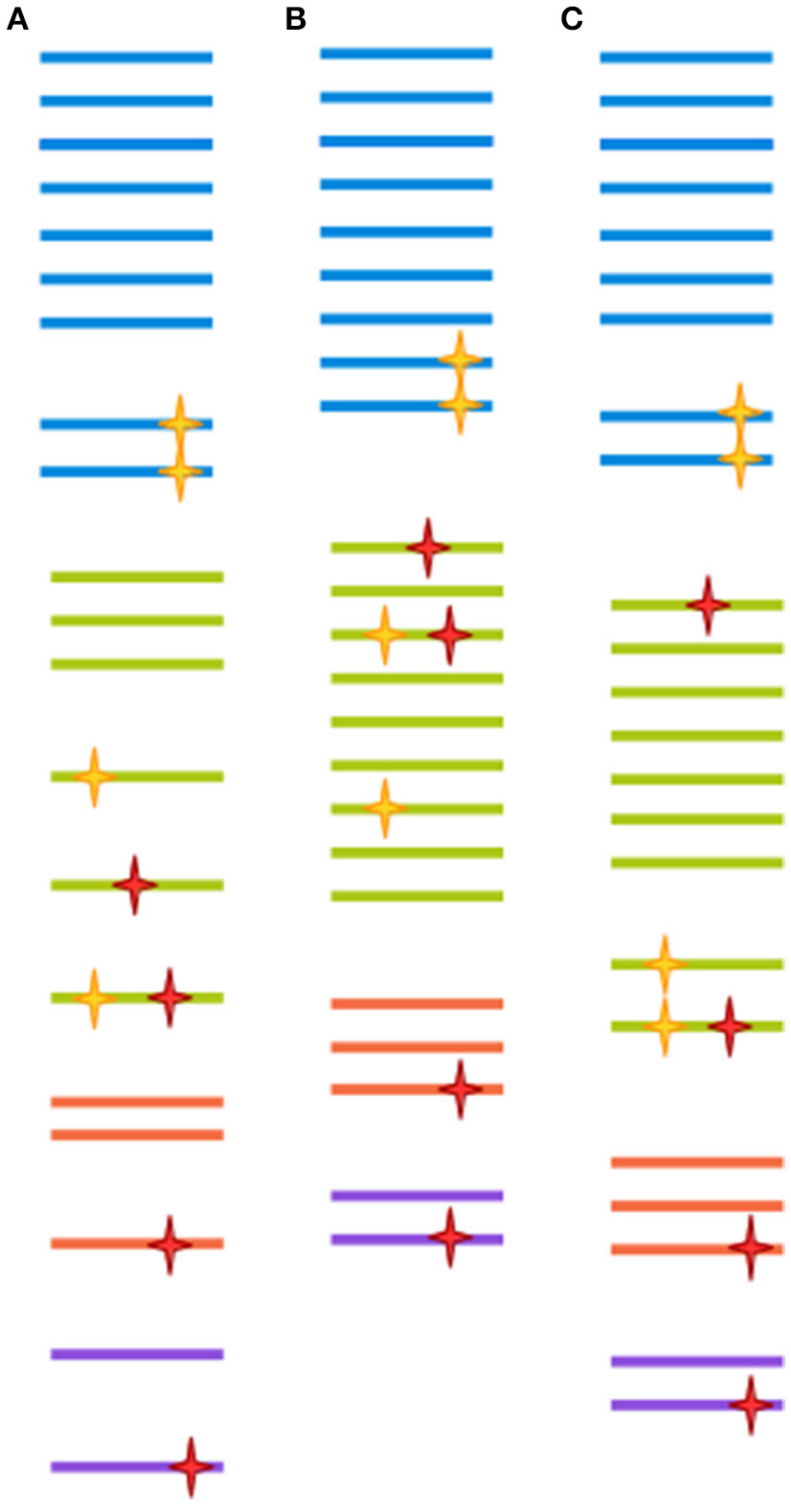
Each clustering approach distorts the OTU composition differently. A hypothetical sample is clustered in three different ways. Each color represents a different clade. Yellow stars represent biological point mutations, and red stars are errors created during amplification and sequencing. The spacing between sequences indicates the inferred clusters. **(A)** Clustering at 100%-identity treats sequences bearing single mismatches as separate OTUs. **(B)** Clustering at 97% identity will remove the effect of amplification/sequencing errors, but will also cluster together sequence variants that represent different clades. **(C)** Modern techniques such as DADA2, cluster-free filtering and minimum entropy decomposition can preserve true diversity while eliminating most spurious mutations. Notice, however, that if mutations are sufficiently abundant (for instance, if they are generated at an early PCR cycle), they might still give rise to a spurious OTU.

Very often, clusters are selected at 97% similarity (Gevers et al., 2005). At lower similarity levels, sequences are unlikely to be derived from the same species, and isolates are unlikely to display 70% DNA-DNA hybridization (a previously common heuristic for determining bacterial species assignment; Stackebrandt and Goebel, 1994; Gevers et al., 2005). However, 97% similarity over the full length of the ~1,500 bp gene doesn't translate directly to 97% similarity over any given region of the gene (Schloss, 2010). Further, as discussed above, a 3% distance doesn't mean exactly the same thing across all packages. Finally, the 97% similarity cutoff is to a large degree arbitrary, since different taxa might have much less of a distance between their tags and still represent ecologically distinct clades (Fox et al., 1992; Gevers et al., 2005). In the field of eukaryotic microbiomics, higher degrees of similarity are often used (Not et al., 2009; Stoeck et al., 2010). This stems from an understanding of the different way taxonomy is applied to eukaryotes as compared to prokaryotes, ie clades with more morphological variety tend to be assigned a more fine-grained classification (Ciccarelli et al., 2006). Some degree of clustering could be deemed necessary when sequencing approaches had much lower throughput, and most reads were likely to be singletons or doubletons (OTU detected by only one or two reads, respectively), which would make statistical comparison between samples very difficult. This, however, is no longer the case. With increased sequence data quality, a 99% cut-off is increasingly common for bacteria as well. The appropriateness of any method is ultimately dependent on the research question being addressed, since OTU clustered at 97% similarity through different approaches have both been shown to recapitulate natural history well, when assessed from a global perspective, and to harbor extreme heterogeneity, when studied at a narrower scale (Koeppel and Wu, 2013; Schmidt et al., 2014). This issue is far from being resolved, as the very concept of species is the subject of much controversy between and within different branches of microbiology (Gevers et al., 2005). Figure 2 illustrates the effect of different clustering approaches on raw sequencing data.

In an attempt to advance the methodological aspect of OTU picking, several approaches have been recently published which attempt to produce biologically meaningful OTU independently of a predefined level of similarity. Each of them has a different strategy to separate the noise introduced by PCR and sequencing from true biological diversity.

DADA2 (Divisive Amplicon Denoising Algorithm 2) initially divides amplicons by considering their abundance distribution (since common reads are more likely to be true sequences) and sequence distance from other reads (since errors are expected to occur at most a few times per read). Then, it uses the clusters generated and the quality scores of bases, produced by the sequencing platform, to calculate a substitution error model conditioned on quality scores for the sequencing run at hand. Finally, it uses this error model to “correct” reads, that is, assign low frequency reads to higher frequency reads from which they could with high probability be derived by substitution (Callahan et al., 2016).

Another strategy, Cluster-Free Filtering (CFF), uses a simpler but conceptually similar approach to figure out which are the true biological sequences in the dataset. Rather than correcting the other sequences, it removes them from the analysis. In addition, the CFF pipeline uses patterns of covariation across samples to infer whether correlated sequence types are most likely derived from different subpopulations or from different operons (or remaining sequencing errors) of the same subpopulation (Tikhonov et al., 2015). The idea in this case is that while different operon copies or erroneous sequences of the same subpopulation will have similar dynamics irrespective of condition, different subpopulations will react differently to different stimuli and their correlation will change between conditions.

Unoise is another denoising algorithm optimized for speed (Edgar, 2016b). It starts by eliminating low-abundance OTU (by default, <4 counts) and then fits an error model in which the larger the distance (substitutions and deletions) between a low-abundance unique sequence and a higher abundance one is, the larger their difference in relative abundance should be, exponentially. Due to this large dependence in abundances, Unoise is best used on full datasets, not individual samples. However, it can become prohibitively slow in large enough datasets, so that a pre-filtering step removing unique sequences with an abundance below, e.g., 10^−6^ can decrease the processing time from days to hours, depending on the evenness of the data. Pre-filtered reads can later be mapped back to the approved centroid sequences to prevent loss of quantitative information. Since DADA2 and Unoise have very different approaches to denoising, they can complement each other and have indeed been shown to produce best results when used in parallel, so that only sequences considered correct by both approaches are accepted as true (Edgar, 2016b).

Minimum-Entropy Decomposition is a denoising method based on the Shannon entropy of each position in an alignment of all sequences. Positions that are peaks in entropy trigger the algorithm to split sequences into smaller clusters of lower entropy. The procedure continues until no cluster exists with a significant entropy peak and a minimum number of sequence reads. Empirically, this approach has been shown to reveal community dynamics that would have been obfuscated by 97% OTU clustering (Eren et al., 2014).

After the OTUs in a study are determined, it is crucial to assign a taxonomic classification to them. This allows the OTUs' dynamics across samples to be interpreted in light of what is known about these taxa from previous studies and, more broadly, allows comparison across microbiomics studies. Unfortunately, there is also no consensus in the microbiology community about how to assign taxonomy to OTU tags. Certain workflows, such as QIIME (Caporaso et al., 2010) and MOTHUR (Schloss et al., 2009), include the classification step. Other softwares are dedicated exclusively to it. For instance, the Ribosomal Database Project (RDP) Classifier uses a naïve bayesian approach to classify sequences based on exact matches of 8-letter words and performs bootstrapping to give probability estimates of the correctness of the assignment (Wang et al., 2007). Another popular approach to sequence classification is the Silva Incremental Aligner (SINA) (Pruesse et al., 2012). SINA uses an initial k-mer based search similar to that of the RDP classifier, but then uses the subset of the reference sequences matched best by the k-mer search to construct a tree representing all unique selected sequences and calculates an exact alignment between the query sequence and the reference candidates. Finally, the sequence taxonomy is assigned as the least common ancestor of the top-scoring alignments.

It is also worth noticing that a classification is only as good as the underlying database. The RDP is maintained by the Center for Molecular ecology at the University of Michigan and it is updated periodically. It includes over three million bacterial and archaeal 16S rRNA genes as well as several thousand fungal 28S and two separate sets of fungal ITS sequences. Another frequently updated database is SILVA, maintained by the Microbial Genomics and Bioinformatics Group at MPI Bremen. It currently contains over five million SSU rRNA gene sequences (16S and 18S) and more than 700,000 LSU sequences (23S and 28S). It also presents subsets of these data including only full length sequences and non-redundant full length sequences. Both the SILVA and the RDP team rely heavily on the work of the Bergey's Manual for taxonomy (Goodfellow et al., 2012) and collaborate with the Bergey Trust. In addition to these, the Greengenes database (DeSantis et al., 2006) combines the NCBI taxonomy with the cyanoDB (Komárek and Hauer, 2013) for a high quality tree of life based on 16S rRNA genes, and it is the default choice for various tools, including the Qiime package and function predictors such as PICRUSt and Tax4Fun (Langille et al., 2013; Aßhauer et al., 2015); however, it hasn't had a new release since 2013, missing the bacterial lineages identified since then. Stand-alone versions of the RDP and SINA classifiers allow the construction of manually curated, personalized databases appropriate to the environment of interest, so the user is by no means limited to the one tool, one database paradigm. A good taxonomic database can also be used to remove spurious sequences, so chloroplast, mitochondria, host rRNA etc., should be included as sanity checks.

Standard approaches generally perform much more poorly for eukaryotes than prokaryotes, due both to more incomplete databases and to a more elaborate taxonomy. Therefore, databases and placement strategies for eukaryotic microbes are still being developed (Lanzén et al., 2012; Guillou et al., 2013; Hu et al., 2015). For well-studied environments of limited diversity, placing OTU directly over a phylogenetic tree is a good strategy for assigning last common ancestor taxonomy to OTU of interest, but this approach is computationally demanding and doesn't scale well for large datasets with high taxonomic diversity (Matsen et al., 2010).

As an alternative to defining and taxonomically classifying OTU *de novo* they can be mapped to reference sequences that have been clustered beforehand (i.e., closed- or open-reference clustering). Examples of such datasets are available in the Greengenes (DeSantis et al., 2006) and Silva (Pruesse et al., 2007) databases. Support for this type of analysis is provided within Qiime, or it can be performed by any blast-like mapping tool, such as Usearch. This approach works well for microbiomes that are well represented in rRNA databases, and makes it easy to add more samples to a comparative study without needing to redo the OTU generation from scratch. For more unexplored environments many reads may lack a close relative and a *de novo* OTU approach is preferable.

Any combination of methods and algorithms chosen to profile community microbiomes have their own intrinsic and unavoidable biases. Even the taxonomy levels considered in each database differ (Balvociūtė and Huson, 2017), which is a major source of variability between pipelines (Siegwald et al., 2017). Which method produces the results closest to the underlying community is difficult to assess and depends on the specific community under study, but being aware of the biases produced by each method is crucial both for method selection and for data interpretation and comparison across studies.

## Unequal sample sizes and data normalization

Multisample microbiomics data is generally summarized as a table of read counts per OTU per sample. These tables are often very sparse, especially for communities with a long tail of OTU belonging to the rare biosphere. The interpretation of counts of 0 is not straightforward, since they may represent the true absence of an OTU or its presence under the detection limit. Moreover, due to e.g., differences in yields between sequencing runs, and unequal representation of samples in pooled sequencing libraries, detection limits will vary between samples.

Due to this lack of clarity on the method's limit of detection, arbitrary approaches are often adopted. Both MOTHUR and Qiime (Schloss et al., 2009; Caporaso et al., 2010) include built-in functions to discard OTU with less than a given number of independent observations, or proportion of reads, or present in fewer than a given number of samples. However, where to set these cutoffs is far from obvious, as it depends on the environment of interest, the sampling scheme and the specific research question. Lundberg et al. (2012) provide in their Supplementary Material an interesting example on how to define these thresholds for a given study. It is also important to consider that eliminating rare OTU or including artifacts may have very large effects on alpha-diversity estimates (discussed below) (McCoy and Matsen, 2013).

This unequal sampling depth makes it often necessary to conduct some kind of normalization. One of the most popular approaches is to divide the counts by the total count of the sample. Doing this breaks the independence of observations, since an increase in the relative abundance of one OTU induces a perceived reduction in all others. A similar approach is using not the total count of reads for normalization, but a fixed percentile of them (Bullard et al., 2010), which should be less sensitive to events such as blooms.

**Figure 3:**
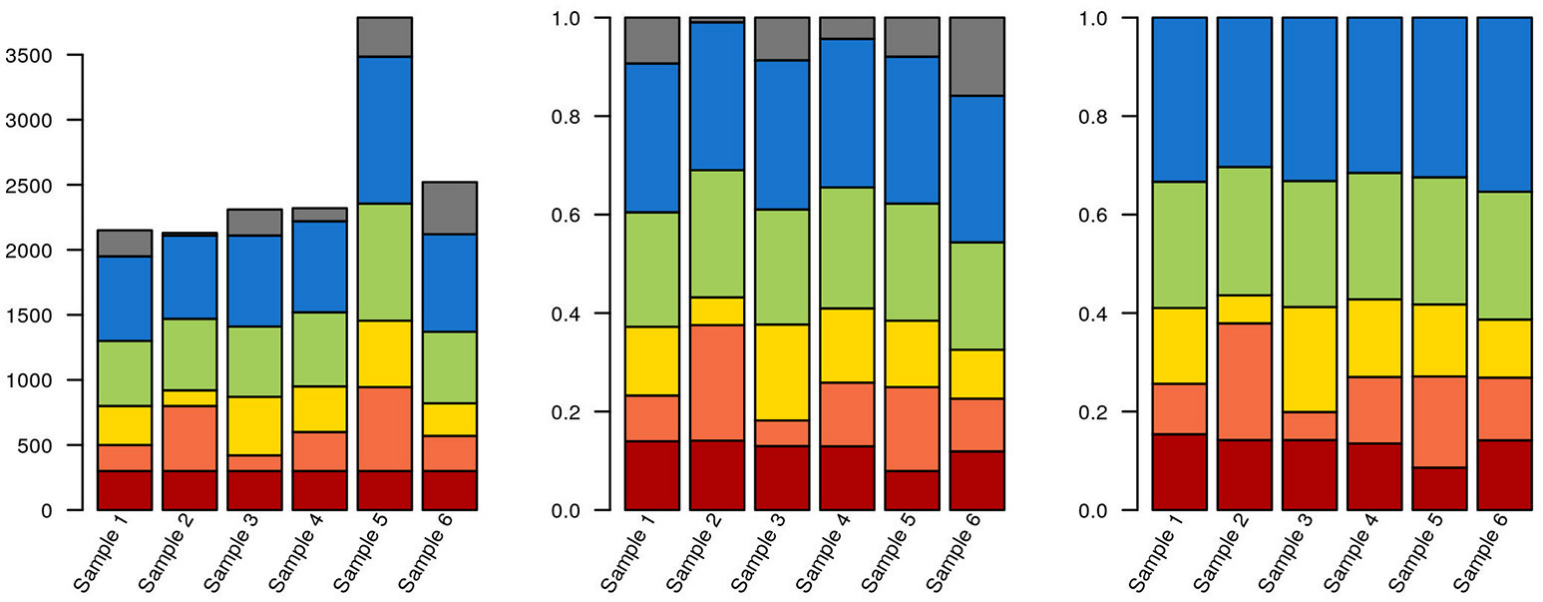
Data selection and normalization affects data representation. The same hypothetical data from a sequencing experiment is depicted in all three panels. Each of the brighter colors represents a clade, and gray corresponds to unclassified sequences. In the leftmost panel, raw data is depicted. The red clade has identical quantities in each sample (300 reads). In the middle panel, the data has been normalized to unity. The blue clade now has the same proportion of reads in each sample (30%). Finally, in the third panel, where only classified sequences are depicted, the green clade has the same proportion of reads in each sample (25%). Also notice that, due to the stacked nature of the bar plots, it isn't necessarily obvious that the green or blue blocks are identical in their respective panels.

**Figure 4:**
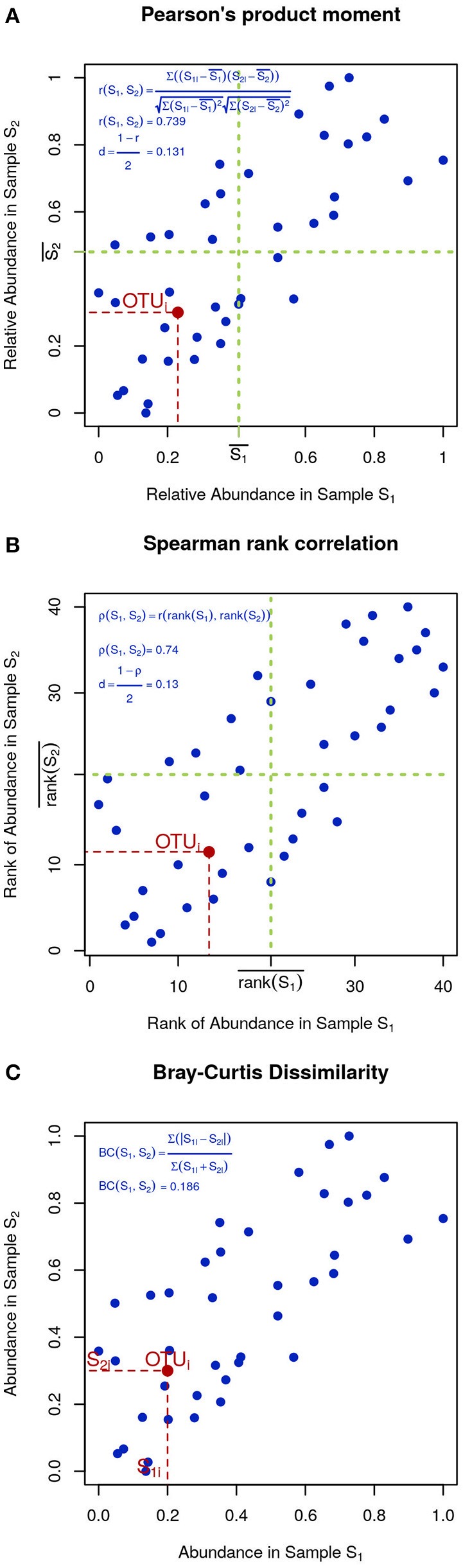
Visual intuition to selected community dissimilarity metrics. In each panel, the same set of OTU (blue dots) is represented in a scatter plot from two highly correlated samples. Pearson's and Spearman's correlations can be intuitively thought as the degree to which the scatter deviates from a straight diagonal line, except Pearson is based on the numeric values of distances **(A)** and Spearman on their ranks **(B)**. Bray-Curtis dissimilarity is displayed in **(C)**.

**Figure 5:**
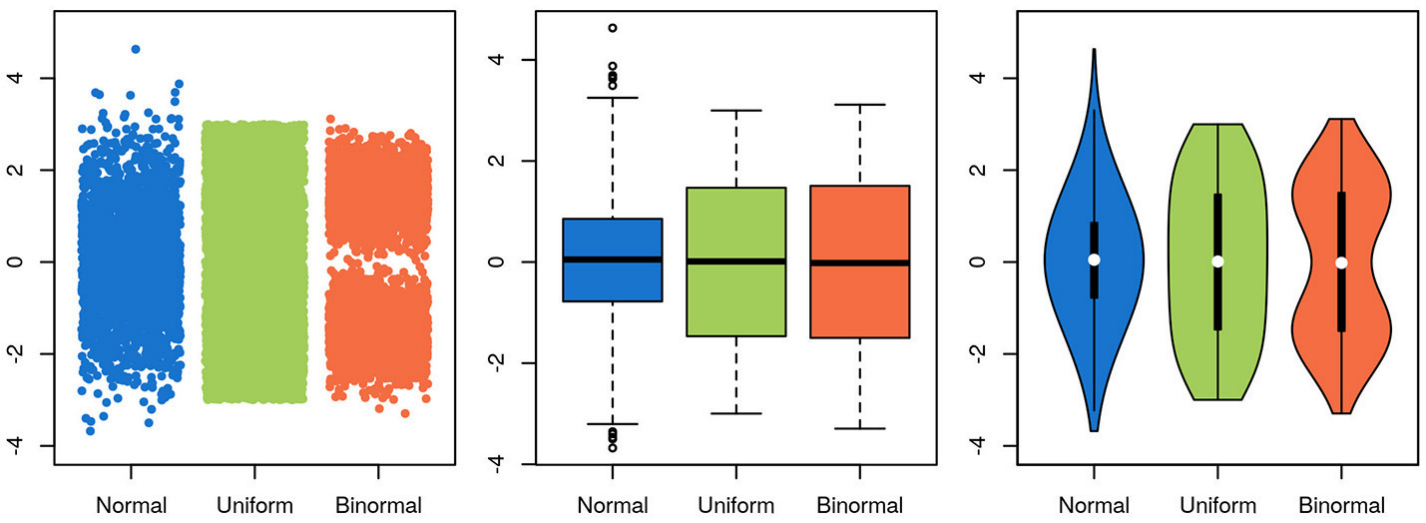
Choosing a visualization technique is a balance between accuracy and clarity. The same three random samples from three underlying distributions are depicted in all panels. A normal distribution is depicted in blue, a uniform in green and a bimodal in orange. In the scatterplot in the leftmost panel, every single datapoint is depicted. While this shows the data accurately, it is harder to estimate the underlying distribution by eye, and adding more points will further obscure the distribution, as the overlap between them increases. Further, the width of the x-scatter, while depicted, is not informative. In the middle panel, the same data is depicted as boxplots. The problem of data overlap is solved by depicting the data in terms of quantiles, but now the binormal and the uniform distribution look very similar. Finally, in the rightmost panel, a violin plot is depicted with the corresponding box-plot on top (boxplot in black, median in white). While not representing the data as fine grained as in the scatter-plot, this depictions represents the data both thoroughly and accurately.

**Figure 6:**
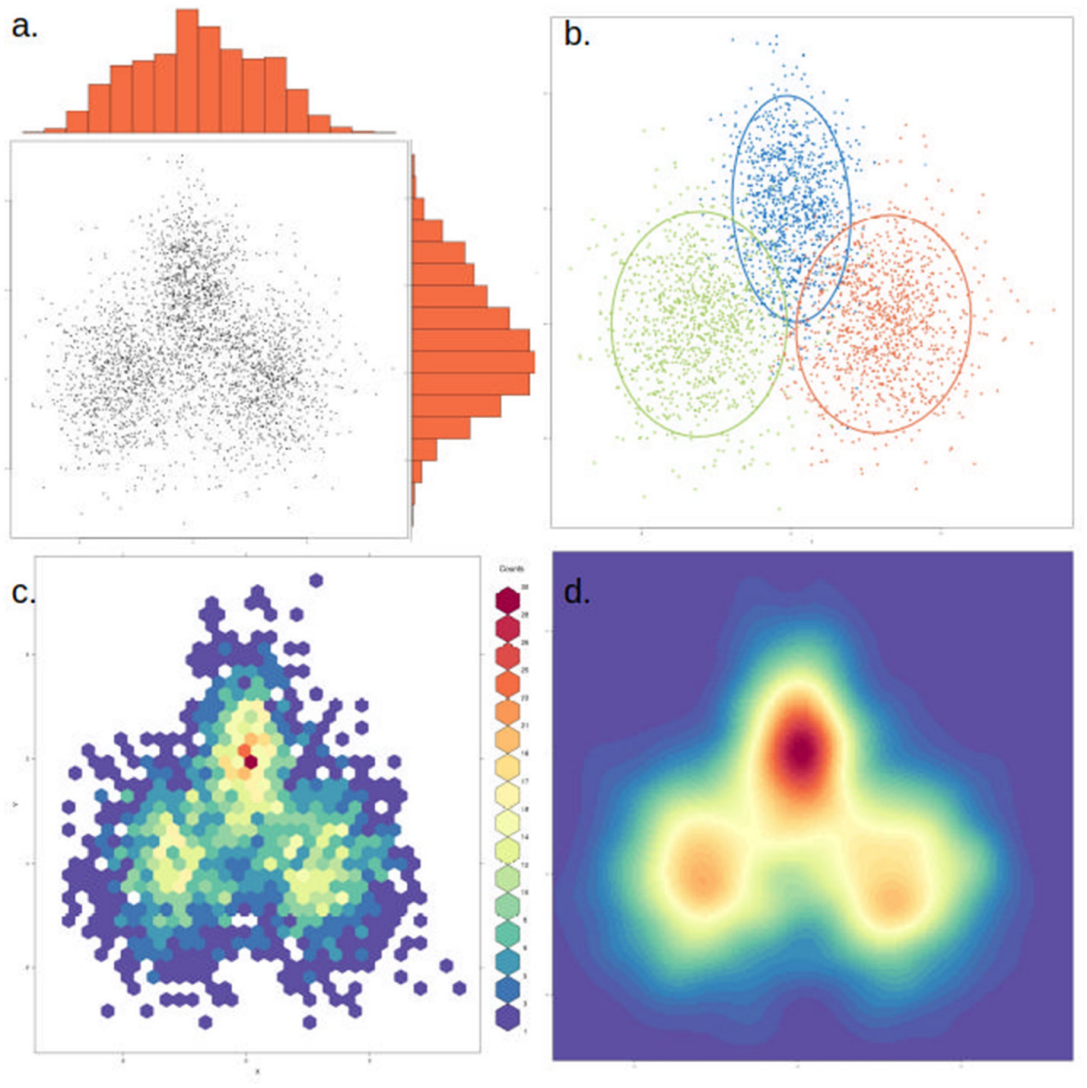
The same data can be represented in different ways to highlight or dampen properties. The same two-dimensional distribution, composed of three overlapping normal distributions with different means and variances, is depicted in each of the four panels. **(a)** Simple scatter plot of the data, with histograms showing the density distribution in each dimension. **(b)** Data points are colored by cluster (representing the underlying distributions) and an ellipse marks the 85th percentile of each distribution. In this case clusters were defined *a priori*, but this could be achieved through various clustering methods. **(c)** The data is binned into hexagonal bins and the color of each hexagon corresponds to the density of points in the bin, as shown in the figure legend. **(d)** Similar to **(c)**, but data is smoothened and interpolated. While clusters are more visible, this approach might also artificially strengthen non-significant clusters.

As an alternative to calculating relative abundances, some authors perform random down-sampling of every sample to the smallest sample size of the cohort. This procedure is a recommended approach for comparing alpha-diversity between samples (Lundin et al., 2012), but downsampling also entails data waste and loss of statistical power for downstream analysis (McMurdie and Holmes, 2014). McMurdie and Holmes also demonstrate that normalizing each sample to 1 doesn't control for overdispersion and, in breaking data independence, increases the rate of false positives. Instead, these authors propose using available packages for mRNA and marker gene sequencing, such as DESeq, edgeR, and metagenomeSeq (Robinson et al., 2010; Paulson et al., 2013; Love et al., 2014). These packages model the dispersion of the data on appropriate models, thereby minimizing both the rate of false negatives and of false positives. Ideally, actual counts can be obtained by multiplying relative abundances by total cell counts, measured by e.g., flow cytometry or microscopy. But even if accurate counts of rRNA gene fragments could be obtained, the variable number of copies of these genes per genome across the tree of life means that this still wouldn't correspond to exact cell counts. Some tools, such as the RDP classifier, can take this into account, at least for well-known lineages. Any choice of normalization will affect how data is interpreted downstream (Figure 3; R code used for generating Figures 3–**6, 8** are provided in Supplementary File 1).

## Estimating diversity within a sample (alpha-diversity)

The term “alpha-diversity” was first defined by Robert Whittaker in 1960 as “The richness in species of a particular stand or community, or a given stratum or group of organisms in a stand” (Whittaker, 1960). In microbial ecology, alpha-diversity is generally understood as the diversity within a single sample or set of replicates. The most naïve way to measure this is observed richness, that is, simply counting how many different OTU are in a sample. However, it is typically impossible to identify every single taxon in a microbial sample, which requires the use of techniques that take into account the incompleteness of the inventory. These can be borrowed from the field of macrobial ecology (Hughes et al., 2001).

One way to estimate the true richness of a sample is to take into account the tail of the species (or OTU) abundance distribution, more specifically the number of singletons (species observed once) and doubletons (species observed twice). This is done by the Chao1 estimator, defined as:

$$\begin{array}{l} S_{est}=S_{obs}+\frac{f_{1}^{2}}{2f_{2}} \end{array}$$

Where *S*_*est*_ is the estimated species richness, *S*_*obs*_ is the observed species richness, *f*_1_ is the number of singletons and *f*_2_ is the number of doubletons. Related to Chao1 is ACE (abundance-based coverage estimator), which considers the ratio not only of singletons and doubletons, but of all OTU observed up to an arbitrary count, most usually 10:

$$\begin{array}{lll} S_{ace} & = & f_{abund}+\frac{f_{rare}}{C_{ace}}+\gamma_{ace}^{2}\frac{f_{1}}{C_{ace}}\\ C_{ace} & = & 1-\frac{f_{1}}{n_{rare}}\\ \gamma_{ace}^{2} & = & \max\left[0,\frac{f_{rare}\overset{10}{\underset{i=1}{\sum }}i\left(i-1\right)f_{i}}{C_{ace}n_{rare}\left(n_{rare}-1\right)}-1\right] \end{array}$$

Where *f*_*abund*_ is the number of OTU above the abundance threshold, *f*_*rare*_ is the number of OTU at or below the threshold, *f*_*i*_ is the number of OTU observed *i* times, *n*_*rare*_ is the total number of individuals in rare OTU, *C*_*ace*_ is a sample coverage estimator and $\gamma_{ace}^{2}$ is the estimated coefficient of variation for rare OTU.

In addition to the number of species in a sample, an important measure of diversity is how even their distribution is. Intuitively, a sample where 10 different OTU each compose 10% of the cells is more diverse than one where one OTU takes up 91% of the sample and the others, 1% each. The Simpson index is a way to quantify this (Simpson, 1949), and it corresponds to the odds that two individual microbes sampled at random will belong to the same OTU.

$$\begin{array}{l} \lambda =\Sigma p_{i}^{2} \end{array}$$

Where λ is the simpson index and *p*_*i*_ is the relative abundance of each OTU *i*.

Another index that measures combined species richness and evenness is Shannon's diversity index, or Shannon entropy. Although originally intended for calculating entropy (uncertainty of information content) of strings of text (Shannon, 1948), Shannon entropy can easily be interpreted in ecology as the uncertainty involved in predicting the species of an individual sampled at random. Mathematically, it is defined as

$$\begin{array}{l} H\prime =-\Sigma p_{i}\ln\left(p_{i}\right) \end{array}$$

From the Shannon index, it is also possible to derive a measure of evenness, Pielou's evenness index, by dividing the observed value of the Shannon index by the highest possible value (that is, that which would be observed if all OTU were present in equal abundance; Pielou, 1966). Mathematically:

$$\begin{array}{l} J\prime =\frac{H\prime }{H_{\max}^{\prime }}\\ H_{\max}^{\prime }=-\Sigma \frac{1}{S}\ln\left(\frac{1}{S}\right)=\ln\left(S\right) \end{array}$$

Where ${H^{\prime }}_{\max}$ is the highest possible Shannon index for a sample with *S* number of OTU.

All of the metrics discussed above give equal weight to each OTU. This would give the same diversity values to a community composed of 10 species from a single genus as it would one composed of 10 different phyla. The phylogenetic diversity of a community can be considered by taking into account the sum of the branches of the phylogenetic tree that includes all OTU in the sample (Faith, 1992), which can also be weighted by the relative abundance of each clade in the sample (Cadotte et al., 2010). The R package Picante can be used to calculate Faith's phylogenetic diversity (Kembel et al., 2010). Building on Faith's original work, other authors have extended measures such as Simpson and Shannon into phylogenetically weighted equivalents (Warwick and Clarke, 1995; Allen et al., 2009). These were later generalized and shown to outperform standard measures at separating healthy from disease-associated human microbiome communities (McCoy and Matsen, 2013).

## Estimating community dissimilarities (beta-diversity)

“Beta-diversity,” as coined by Whittaker (1960), is “The extent of change of community composition, or degree of community differentiation, in relation to a complex gradient of environment, or a pattern of environments.” In other words, beta-diversity is the degree to which two samples are different. This is a rather different issue than within-sample richness and evenness, and can be measured in many different ways.

The choice of beta-diversity metric can have important consequences to subsequent analyses, such as clustering and ordination. This is partially due to the interplay between distance metrics and normalization techniques, which can widen or reduce the apparent distance between samples (Figure 3).

A true distance metric is one that is always positive, in which the distance between a point and itself is 0, the distance between A and B is identical to the distance between B and A and the sum of the distance between A and B and between B and C is no greater than the distance between A and C. This last assumption is the one that often fails for other dissimilarity measures. The appropriate metric for a study might depend on the size of the effect of interest and on the depth of sampling.

The most widely known true distance metric is the euclidean:

$$\begin{array}{l} d\left(S_{1},S_{2}\right)=\sqrt{\sum {\left(S_{1i}-S_{2i}\right)}^{2}} \end{array}$$

Where *S*_1_ and *S*_2_ are two samples and *S*_1*i*_, *S*_2*i*_ are the abundance of OTU *i* in samples *S*_1_ and *S*_2_, respectively.

However, the euclidean distance requires very large effect sizes for statistical significance (Kuczynski et al., 2010) and doesn't perform well in datasets with many zeroes. A more appropriate metric is thus Jensen-Shannon's, a symmetric version of the Kullback-Leibler divergence. In Kullback-Leibler, the distance between *S*_1_, *S*_2_ is:

$$\begin{array}{l} KL\left(S_{1},S_{2}\right)=\sum S_{1i}\times ln\frac{S_{1i}}{S_{2i}} \end{array}$$

Thus, Kullback-Leibler is not applicable for 0-rich datasets. However, since Jensen-Shannon's compares samples *S*_1_ and *S*_2_ to their average, the problem of 0's disappears:

$$\begin{array}{l} JS\left(S_{1},S_{2}\right)=\frac{1}{2}\times KL\left(S_{1},\frac{S_{1}+S_{2}}{2}\right)+\frac{1}{2}\times KL\left(S_{2},\frac{S_{1}+S_{2}}{2}\right) \end{array}$$

This formulation also automatically satisfies the other requirements for a distance metric.

In microbial ecology it is also common to use correlation coefficients, such as Pearson's product moment (Figure 4A):

$$r\left(S_{1},S_{2}\right)=\frac{\sum \left(S_{1i}-\overset{-}{S_{1}}\right)\left(S_{2i}-\overset{-}{S_{2}}\right)}{\sqrt{{\sum \left(S_{1i}-\overset{-}{S_{1}}\right)}^{2}}\sqrt{\sum {\left(S_{2i}-{\overset{-}{S}}_{2}\right)}^{2}}},$$

To minimize the influence of noise, other researchers prefer Spearman's rank correlation, which is identical to Pearson's except that instead of the measured values, their ranks are used (Figure 4B). Finally, Bray-Curtis dissimilarity, while not very sensitive, is appropriate for 0-inflated datasets (Figure 4C):

$$\begin{array}{l} BC\left(S_{1},S_{2}\right)=\frac{\sum |S_{1i}-S_{2i}|}{\sum \left(S_{1i}+S_{2i}\right)} \end{array}$$

An alternative to OTU-based distances is to use phylogenetic distances. While these approaches also require several non-trivial choices, such as the underlying phylogenetic tree and the placement of OTU in it, evolutionary distances are often more biologically meaningful, not least because phylogenetic relatedness is often associated to trait conservation (Martiny et al., 2015). As is the case for OTU-based metrics, using a quantitative or qualitative approach to community comparison can lead to very different results (Lozupone et al., 2007). This can be ameliorated through an appropriate weighting procedure, such as generalized Unifrac (Chen et al., 2012). Recent work by Schmidt and colleagues does a thorough review of commonly used distance metrics and proposes new taxonomic and phylogenetic distances based on co-occurrence networks (Schmidt et al., 2016).

Different approaches to community dissimilarity, such as OTU-based vs. phylogenetic, may highlight different aspects of the community and its functioning. It can therefore be useful to combine these different analyses to gain deeper insight into the system under study.

## Visualizing high-dimensional data

To be useful, a graphical representation of data must be more readily interpretable than the raw data. This is usually achieved by decreasing the level of detail in the data. A boxplot, for instance, contains much less information than a scatter-plot of the same data, but is often more easily apprehendable. In this specific case of unidimensional data with many data points, a balance between information-richness and interpretability can be achieved through the use of violin plots (Figure 5). In other cases, information is added to a plot, for instance through the use of color indicating data density or outlines highlighting groups of interest. Care needs to be taken then to not induce false conclusions. While the reader may be aware of which aspects of the graph aren't strictly informative, it is difficult to not be led, at least subconsciously, by these elements.

A simple visual inspection of the data is often the first step of any analysis. In microbiomics, this translates into plotting OTU abundance per sample. Since the number of OTU is generally incompatible with thorough plotting, they are often aggregated at higher taxonomic levels. It is usually recommendable to make these plots at various taxonomic levels, since community composition could, for instance, be stable at the phylum level but highly dynamic at the family level or, alternatively, highly stable for all but a few families which drive large phylum-level differences. A useful tool to avoid these constraints is Krona, which makes hierarchical interactive pie-charts representing several taxonomic levels at the same time (Ondov et al., 2011). However, when analysing a large number of samples, pie charts can make comparison across samples unintuitive, since there is no structured spatial organization of the data.

Stacked bar plots and line plots (stacked or not) are good alternatives for representing large numbers of samples. When comparing temporal series or data that is physically structured, line plots have the advantage of preserving the (temporal or geographical) distance between samples. On the other hand, the very existence of a line connecting points suggests that data changes smoothly across that interval, which may in fact be far from true. Barplots, on the other hand, preserve the discrete nature of data collection, but generally only display sample labels on the x-axis, making them less informative. Finally, authors must decide how to present the data in their bar plots, whether to normalize each sample to 1, or normalize the presented portion of the data (for instance, only OTU which have taxonomy at least at the domain level) to 1, for example (Figure 3). Each of these choices will highlight a different aspect of the data and must be clearly stated.

Since the human mind cannot process images in more than three dimensions, one of the main challenges in dealing with the vast number of OTU and/or samples in a microbiomics study is condensing the information into two- or three-dimensional spaces. A very good overview of techniques to achieve this was written by Paliy and Shankar (2016). One of the oldest and most common methods to achieve this is principal component analysis, or PCA (Ringnér, 2008). In it, variables are treated as axes in a euclidean multidimensional space and the first principal component is by definition placed on the direction representing the largest variation of the data. The second component is placed in the direction orthogonal to this that explains the largest amount of the remaining variation, and so on. The first few components often explain a large amount of the variation, allowing a visual inspection of the distance between samples in two- or three-dimensional space. Furthermore, the percentage of the variation explained by each axis indicates whether there are dominant drivers present or not. However, the euclidean distance is seldom appropriate for microbial data, since pairs of samples with many common counts of 0 are given a short distance. Bray-Curtis dissimilarity, in contrast, only considers OTU present in at least one of the samples being compared.

To avoid the constraint of the euclidean distance, principal coordinate analysis (PCoA) can be used together with any dissimilarity matrix, such as Bray-Curtis or UniFrac, which are more appropriate for microbiome data. Another conceptually similar strategy is correspondence analysis (CA), where rather than maximizing the percentage of variance explained by each axis, the correspondence between rows and columns in the matrix is optimized. In the case of UniFrac, there are also specific methods developed for it, such as edge PCA, which directly selects high variability lineages as axes on the PCA, allowing a direct phylogenetic interpretation of the resulting plot (Matsen and Evans, 2013).

To assist in the graphical interpretation of a PCA or PCoA, it can be useful to plot each of the measured variables against the two main components. An extension of this that facilitates comparing separate PCA is the circle plot, which focuses only on the variables measured and how they relate to each other (independent, positive or negative correlation). PCA clusters can also become difficult to visualize if there are too many data points. In this case, an alternative to plotting each point is to use a density cloud, where the number of points per area unit is shown with the use of color. Finally, it can be useful to highlight clusters with a visible elliptic contour covering, for instance, 1–2 standard deviations from the cluster's center (Figure 6). This is useful to highlight *a priori* expected clusters and how they compare to the actual results, or in cases where clusters are poorly visible in just a few dimensions. Nevertheless, when interpreting plots containing contours it is important to discern which clusters are clearly visible and which are merely highlighted. It is also possible to calculate how many axes are shared from two or more PCA analyses, using the technique of common principal component analysis (CPCA).

Unlike these techniques, in multidimensional scaling (MDS), the number of dimensions to which the dataset should be reduced is chosen a priori and the algorithm finds the distribution of objects in the lower-dimension space that best corresponds to their distances in the full dimension, while also calculating a stress function representing the amount of the distortion between the true distances and the distances in the reduced space. If using euclidean distances, i.e., a “classical MDS,” the result is identical to a PCA. However, other true distance metrics can be used in metric MDS, and non-metric MDS is an extension of this technique using the ranks of distances rather than their values. Clarke (1993) includes many interesting practical considerations in the interpretation of MDS plots.

Once relevant clusters are determined, either through exploratory techniques or through the use of previous knowledge, linear discriminant analysis (LDA or its multisample counterpart MDA) can be used to define which linear combination of quantitative descriptive variables best separates these clusters. If using clusters found by a dimension-reduction approach, however, it is crucial that the LDA is performed on independent data. The model generated by the LDA can later be used to partition new data into one of the known clusters. LDA will fail if any of the clusters has too few data points, if their variance is not independent from their mean and in the presence of categorical variables or significantly non-linear interactions between variables.

It is often not clear what the main driver of the community over the gradient is, or even how many overlapping gradients there are. These are the cases where exploratory methods are most needed, but also where the biases of each method can most affect the biological interpretation of results. For instance, the horseshoe effect, where sparse matrices driven by a single dominant gradient assume an arch-like pattern when submitted to PCA or CA, may mask other, more subtle gradients, and detrending techniques used to eliminate this effect often erase true patterns (Kuczynski et al., 2010). In datasets with many overlapping gradients, an NMDS will often produce a clearer overview of the data distribution than methods that don't limit the number of dimensions (Paliy and Shankar, 2016). It is therefore recommendable to try a variety of different approaches and retain not merely those which explain the largest proportion of the variation in the dataset, but also those that propose underlying biological mechanisms amenable to further investigation.

Another popular method for visualizing data clusters, specially when there are more variables than samples, is through the use of heatmaps, often associated to a dendrogram. The main problem with this practice is the use of the red-green color scale, which is inaccessible to up to 8% of the male population (Simunovic, 2010). This is however easily by-passed through the use of a yellow-blue color scale. Another problem is the associated use of dendrograms. Since the human mind gives much more emphasis to distance than to associated lines, people tend to perceive data rows or columns that are side-by-side as more similar than those with a smaller branch length, but further apart. This is unavoidable in the use of trees, and something that has to be kept in mind when inspecting one.

## Hypothesis testing

In addition to visually inspecting the data, it is important to have statistical tests to assess the plausibility of proposed hypotheses. Typical questions that a researcher might ask from these data are: does the microbial community cluster according to predefined sample groups, eg patients and healthy controls? Does the distribution of the community reflect the underlying contextual parameters, eg physicochemical environmental data? What component of the environmental or patient data corresponds to the largest shift in the microbial community? Conversely, what components of the microbial community correspond to the largest shift in health or environmental markers?

For instance, it might be important to assess whether a *priori* groupings of samples, such as different environments or treatment groups, correspond indeed to statistically different microbial communities. Most researchers are familiar with one- and two-way analysis of variance (ANOVA/MANOVA). However, due to the non-normality of most microbial data, non-parametric versions of these tests are needed. Kruskal-Wallis' *H*-test, also known as “ANOVA on ranks” is suitable when there are only two sample groups. For multiple comparisons, non-parametric MANOVA is often termed PERMANOVA, since permutations are used to assess significance. ANOSIM is a similar test, which assesses whether ranks of distances of objects within *a priori* defined classes are smaller than between those classes. Closely related to these tests, linear discriminant analysis (LDA) tests whether groups of samples are significantly different on multiple axes and then attempts to find one axis that optimally discriminates the groups. However, LDA requires that the groups' variance is independent from their mean, which is often not the case in microbiomics data.

There are also several tests available that assess how similar two matrices are. This is the mathematical equivalent to visually assessing the likeness of two PCAs (e.g., one based on OTU and one based on metadata) to say whether they likely reflect related phenomena. For instance, canonical correlation analysis (CCorA) tries to find the linear combinations of variables in two datasets that provide the maximum correlation between them. The non-parametric extension of CCorA is called BIOENV and is deemed more suitable for ecological data (Clarke, 1993; Clarke and Ainsworth, 1993). In Procrustes analysis, the same set of objects (e.g., samples) placed on different spaces (e.g., biological domains or metabolites) are moved, rotated and scaled to minimize the sum of distances between pairs of corresponding objects. A conceptually similar test is Mantel's, which calculates the correlation between two distance matrices and assesses significance by permutation.

When it is clear which are the explanatory variables and which are the response variables, methods can be constrained accordingly. Redundancy analysis (RDA) extracts and summarizes the extent of variation in a response dataset which can be accounted by an explanatory dataset. Likewise, canonical correspondence analysis (CCA) maximizes the correspondence between rows and columns in a table, constrained to the explanatory variables.

If one variable overwhelms the effect of all others, as can be the case in intervention studies in which all treated samples are clustered together and apart from the non-treated, a principal responses curve (PRC) can be used (van den Brink et al., 2009). This approach is also useful if an overlap of many potentially interacting gradients makes the visual interpretation of an RDA or CCA plot impossible. Other approaches with the same goal, such as partial least-squared regression and canonical inertia analysis are thoroughly discussed by Le Cao et al. (2009).

None of the strategies discussed here can distinguish correlation from causation, except perhaps in intervention studies. More importantly, clusters and gradients produced along artificial axes do not necessarily correspond to any underlying biological effect. From a mathematical perspective, variables of different types (e.g., metabolomics vs. microbiomics) will often have different variance-to-mean characteristics, which requires appropriate data transformation (Paliy and Shankar, 2016). New methods for testing hypotheses based on high-throughput data are still being developed, and understanding their strengths as well as their assumptions is a crucial and challenging issue for microbial ecologists. Detailed descriptions, assumptions, limitations and test cases of many popular statistical methods for ecological research can be found in the GUSTAME server (Buttigieg and Ramette, 2014), and in the review by Paliy and Shankar (2016).

## Assessing the roles of specific OTUs in the community

In many cases, it isn't enough to determine how contextual data interact with the microbiome at the community level. In addition, it may be important to determine which organisms contribute most to the community differences. Similarity percentages breakdown (SIMPER) measures the contribution of individual OTUs to Bray-Curtis dissimilarities between sample groups (Clarke, 1993). In other cases, even if the total community isn't significantly different, a subset of OTU might still display significant changes in abundance, such as pathogens or taxa with unusual metabolic capabilities. This is important in the development of diagnostic tools, environmental surveillance strategies and generally for generating testable hypotheses.

In some cases, identifying differentially abundant OTU can be straightforward. For instance, after detecting conditionally rare taxa, Shade et al. (2014) directly calculated which fraction of the distance between communities could be attributed to these specific OTUs. Often, however, there is no clear *a priori* choice of OTU to analyse, and specific methods have to be applied. From a mathematical perspective, there are three main challenges to identifying differentially abundant OTU: the variance of each OTU is not independent from its measured value (heteroskedasticity), most OTUs are below detection limit in most samples (0-inflation, or sparsity) and, due to normalization procedures, the observed value for each OTU in a sample depends on the others (non-independence). Additionally, different statistical tests perform quite differently in cases close to the detection limit, with e.g., the *t*-test failing when the count on either sample under analysis is 0, but Fisher's test performing as expected (Bullard et al., 2010).

Metastats, released in 2009, deals with sparsity by separately considering sparsely sampled OTU using Fisher's exact test (White et al., 2009). Instead of assuming data normality, a nonparametric *t*-test is used, with multiple testing correction performed by calculating the false discovery rate.

Many tools initially developed for RNA-sequencing data can also be used for microbiome studies (Jonsson et al., 2016). Released in 2010, edgeR explicitly models the underlying distribution of each feature (e.g., gene or OTU) as a negative binomial distribution, using an empirical Bayes procedure and conditioning each OTU's variance on their abundance (Robinson et al., 2010). Several other tools were released since then that model the distribution of each OTU using similar procedures. One of the most popular is DESeq2 (Love et al., 2014). In it, information is shared across OTU and it is assumed that OTU with similar abundance will have similar dispersions. This assumption is however over-ruled when the observed variance is more than two-fold different from the mean variance. DESeq2 also considers that noise is greater when counts are low, and is more aggressive in its variation shrinkage approach for low-abundance OTU. Significance of differentially abundant OTU is assessed via a Wald test and multiple testing correction is performed via Benjamini-Hochberg, but the false negative rate is minimized by previous removal of low abundance OTU (whose likelihood of being significantly differentially abundant is, in any case, low). DESEeq2 also includes a tool for making the variance of each OTU independent from its mean (regularized log normalization), a formal requirement for many of the machine learning and ordination methods discussed in this review.

As an alternative to methods such as edgeR or DESeq2 that depend on the negative binomial distribution, SAMSeq (Li and Tibshirani, 2011) was developed as a non-parametric method. SAMSeq conducts Mann–Whitney test on multiple resampling of the data to account for different sequencing depths. It has been reported to give fewer false positives than the negative-binomial tests but has low sensitivity in case of small sample sizes.

A Bioconductor package explicitly aiming at modeling OTU count data was released in 2013 as metagenomeSeq (Paulson et al., 2013). This package introduces two novelties. Firstly, instead of normalizing counts by the total sum of each sample, a percentile cut-off is used. This percentile is chosen automatically by selecting the highest percentile after which there is a large instability between expected values and observed values, suggestive of PCR biases. In addition to this, since microbiomics data is generally much more sparse than RNA sequencing data, a different distribution was chosen to model the data, namely a zero-inflated Gaussian. However, posterior work showed that the zero-inflated Gaussian has a higher rate of false positives than negative-binomial based approaches, and recommended either edgeR or DESeq2 as best practices (McMurdie and Holmes, 2014). These packages and others can be easily used in R in combination with other microbiomics tools through the wrapper package PhyloSeq, which also includes extensive documentation of its features (McMurdie and Holmes, 2013).

The linear discriminant analysis effect size method (LEfSe; Segata et al., 2011) takes a different approach by combining standard statistical tests with the usage of previous biological knowledge in its search for markers. After a first round of feature selection through Kruskal-Wallis' sum-rank test, which identifies OTU differentially abundant between conditions, LEfSe uses pairwise Wilcoxon's tests to discard OTU whose differential abundance isn't consistent across sub-conditions, a step intended to remove spurious correlations. Since these two tests are non-parametric, the possible non-normality of the data is not an issue. Finally, it uses linear discriminant analysis to estimate the effect size of each differentially abundant OTU, an important step in biomarker discovery, as even a highly statistically significant marker is unlikely to be driving environmental or host phenotypic changes if its effect size is too small. The particular setup of LEfSe emphasizes the need to address a range of relevant conditions within the characteristic under study, and is therefore an interesting example of a computational method driving study design.

## Community dynamics and network reconstruction

While assessing the relationship between microbial communities and environmental parameters or treatments will always be of fundamental importance, there is mounting evidence that, within a given ecosystem, interactions between taxa play a more important role in driving community dynamics than environmental forcing (Gilbert et al., 2012; Lima-Mendez et al., 2015).

**Figure 7:**
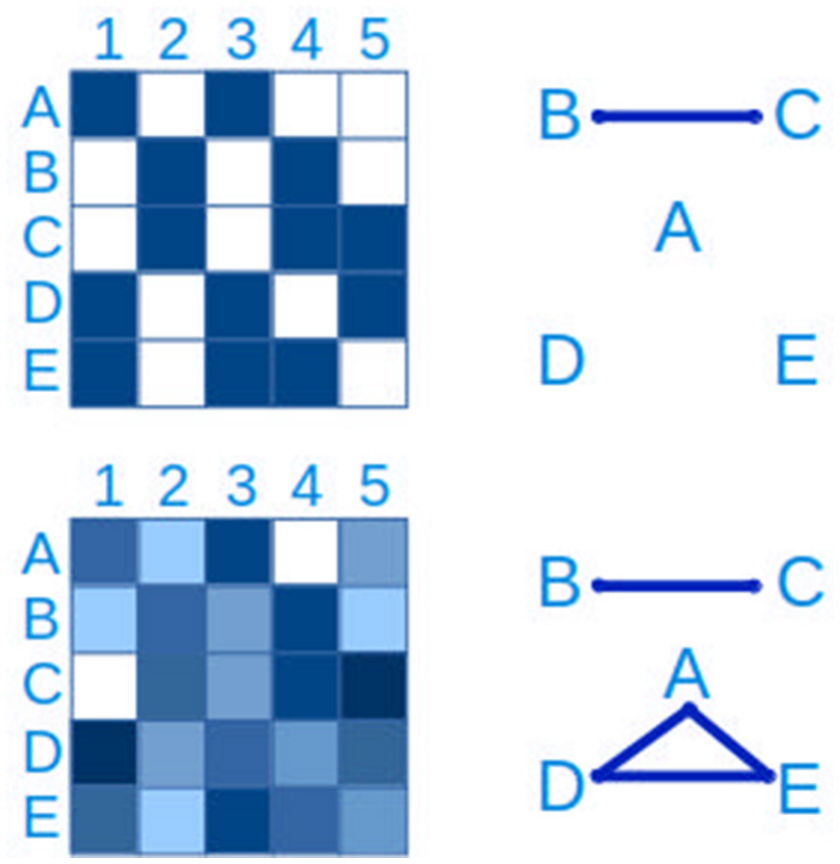
Connections between taxa can be inferred from checkerboard patterns. The same data is represented on the left side. In the upper panel, a presence-absence table of clades A–E in samples 1-5 is represented, where blue is presence and white is absence. From this data it is possible to infer a connection between clades B and C. In the lower left panel, the presence-absence table has been replaced by a quantitative table, with a lower detection limit, more typical of microbial ecology studies. In this case, in addition to the connection between clades B and C, a three-way connection between clades A,D and E is inferred.

The most basic approach to hypothesizing interactions between microbial populations is through pairwise relationships, either as presence/absence (“checkerboard patterns”) or through quantitative measures (Figure 7). The latter generally relies on measures of correlation such as Spearman's and Pearson's, while the hypergeometric distribution is appropriate for binary data (Chaffron et al., 2010; Freilich et al., 2010). WGCNA (Langfelder and Horvath, 2008) is a useful R package to create, analyse, compare and visualize correlation networks.

These simple correlation approaches are hampered by limited sampling depth and the ensuing compositionality of the data, which induces spurious correlations. SparCC is a tool built with this caveat in focus, and by-passes it by including the calculation of effective sample-size in its interaction estimation (Friedman and Alm, 2012). SparCC assumes that every OTU is present in every sample, but that they are often below detection limit. Therefore, OTU expected to be very rare and seldom present should not be included in its estimates. However, others have shown that increased rarefaction of data (leading to increase in proportion of 0 count OTU) greatly increases the rate of false positives for all methods tested (Weiss et al., 2016).

Regardless of the procedure adopted, the underlying hypothesis is that, if there is an interaction between two species, and given similar environments with similar resources, these two species will co-occur more likely than expected by chance if their interaction is beneficial (mutualism or commensalism) and co-occur less likely than expected by chance if their interactions is prejudicial (competition or amensalism). However, two of the most important types of interactions in natural systems, predation and parasitism, are beneficial to one of the parts (the predator or parasite) and prejudicial to the other (the prey or host), complicating the ecological interpretation of co-occurrence patterns. Furthermore, given the intricacies of microbial metabolism, it is seldom clear if a species is excluded from a niche due to negative interactions with other organisms or due to environmental constraints. Nevertheless, mapping pairwise correlations can be a useful first step in developing an interaction hypothesis.

Other available approaches do not depend on monotonic correlations. Maximal Information Coefficient (MIC) (Reshef et al., 2011) is a non-parametric approach designed to detect associations and to give similar scores to associations with similar noise levels, regardless of their shape (linear, exponential, periodic etc.). Intuitively, this is achieved by plotting the abundance of OTUs against each other, pairwise, and over each plot defining a grid which splits the sections of the graph that contain data from those which do not. Mutual information—a measure of the predictability of two variables in relation to each other—is then calculated for each section of the grid. The MIC algorithm penalizes overly complex relations by decreasing the score according to the number of partitions in the grid.

Since, in the typical case, thousands of correlations and anticorrelations will be tested, the significance of any association has to be tested and subjected to multiple testing correction. This is often done by randomizing the interaction network and calculating the distribution of scores. It is however still not clear what the correct randomization procedure is for this type of data (Faust and Raes, 2012). One alternative to reduce the rate of false positive inferences is to combine different approaches and keep only links supported by multiple sources of evidence. A tool for doing this was introduced by Faust et al. (2012) and is alternatively called CoNet, Reboot or CCRePe, depending on its implementation. However, in further work, the same authors used CoNet as only one of the elements in a more elaborate ensemble approach with superior results (Weiss et al., 2016).

The pairwise interactions inferred by the techniques described above can be used to build networks where each OTU or measured environmental parameter is a node and interactions between them are links. In addition to being a rich representation of interactions between particular nodes, properties of the network itself can contain information about the system. For instance, microbial networks are generally modular, scale-free and have short average path length (Faust and Raes, 2012). How these mathematical properties translate into biological properties is still open to debate. It is not clear, for instance, whether a node with a high degree (i.e., linked to many nodes in the network) represents a keystone clade whose demise would severely perturb the entire system, or whether the levels of redundancy and plasticity in biological systems are enough to functionally replace these hubs without much propagation of perturbation. In the case of bacteria in particular, not only does the community present a certain level of plasticity, but single populations and even individual cells can dramatically alter their life strategy in response to disturbances, decoupling to a large extent a community's taxonomic composition from its functional profile (Shade et al., 2012; Comte et al., 2013). Network properties also interact with community characteristics such as richness and evenness, and often have opposite effects in the resulting resistance and resilience of the community to perturbation, so that broad natural laws of community stability might be impossible to obtain (Shade et al., 2012).

A powerful approach to gain insight into the internal mechanisms of a natural microbial community is sampling a time-series with appropriate intervals and length, and using techniques such as Local Similarity Analysis (Ruan et al., 2006; Steele et al., 2011) or auto- and cross-correlation (Fuhrman et al., 2006; Gilbert et al., 2012; David et al., 2014). If a system has an intrinsic periodicity, such as annual cycles, a few full cycles should be included in the study to separate recurring patterns, random fluctuations and system drift (time decay; Gilbert et al., 2012; Kara et al., 2013). Also important is to consider that different processes might take place at different rates, corresponding to one or more sampling intervals or, conversely, that associations that are significant in the short term can be irrelevant at longer time-spans (Steele et al., 2011; Needham et al., 2013).

**Figure 8:**
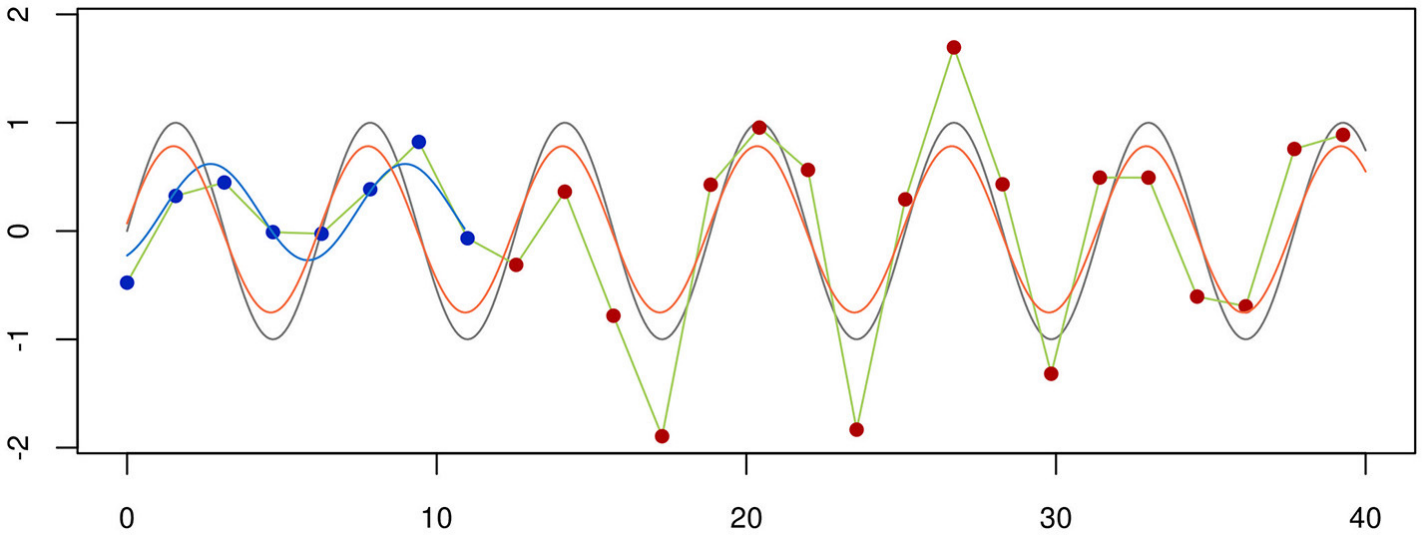
Temporal decay is dampened in longer time-series. The gray line represents an oscillator, e.g., a microbial community subjected to strong seasonal variation. Each point is one sample, representing the intrinsic oscillation of the community plus stochastic deviations from it. Simply connecting the points (green line) doesn't give any mechanistic insight. A first mechanistic hypothesis can be generated from a few cycles (blue dots, blue line), but it is significantly worse than a more complete temporal series (all circles, orange line).

Strong seasonal recurrence has been reported in several sites, with the rate of interannual decay declining with the length of the time-series (Gilbert et al., 2012; Cram et al., 2015). Due to seasonality, the time-frame which is relevant for most free-living microbial assemblages are those which are one-year apart, i.e., in the same season. Stochastic factors mean that there is significant loss of signal from one year to the next. However, environmental and biological constraints maintain community variation within certain boundaries (Figure 8). In addition to recurrent and linear (time-decay) patterns, dramatic but rare events can occasionally also be observed in long time-series (Gilbert et al., 2012; Vergin et al., 2013; Lindh et al., 2015).

Local Similarity Analysis is a strategy optimized for time-series data and non-linear interactions (Ruan et al., 2006), and is available as the stand-alone package eLSA (Xia et al., 2013). It is robust to data sparsity and was evaluated by Weiss et al. (2016) to be the overall best approach for time-series data. They note, however, that the frequency of sampling plays an important part in the algorithm's accuracy. While eLSA can fill in missing data by interpolation, it is not clear to what extent this can mask or induce spurious correlations.

Weiss et al. (2016) further noted that none of the strategies currently available is sufficiently robust. Even the choice of sequencing technology was observed to significantly affect the output of network inference algorithms. Experimental validation is therefore crucial, but can be very hard to conduct, especially if the interactions do not involve physical contact of the cells. Carefully considering the sampling procedure and adapting it to the needs of the network inference algorithm is therefore recommended. Furthermore, it is generally still not possible to accurately predict interactions involving one or more OTU which is present in only a small fraction of the samples, so computational time and statistical power can be saved by removing rare OTU (present in less than 30–60% of samples; Weiss et al., 2016) before network inference.

A very different approach to generating predictive models for OTUs based on environmental data and/or other OTUs is through artificial neural networks (ANN) or Random Forests. Briefly, an ANN is a layered series of computing units, analogous to neurons in a real neural circuit. Raw data is fed to an initial layer and is then relayed non-linearly through each layer of the ANN. At each layer, each computing unit receives data from each unit of the previous layer, and performs a weighting procedure to its input and then another non-linear operation. At the output, a classification or numeric prediction is made. Despite very promising results (Larsen et al., 2012), this approach has not been widely adopted by the field. A more thorough discussion of neural networks and applications to computational biology can be found in Angermueller et al. (2016).

Random Forests, on the other hand, are machine learning strategies based on decision trees. A decision tree starts with a table of pre-classified data. Based on that, it determines decision criteria for classifying new data. In addition to that, a decision tree can be used to fit, e.g., linear models to each partition, if the data of interest is quantitative. Random Forests are an extension of decision trees where several random subsets of the total data are given as input to different trees (thereby creating a forest of decision trees). This increases the robustness of the prediction and allows the estimation of classification accuracy based on the training data. For datasets with a large number of parameters, improved predictions can sometimes be achieved with a pre-selection criterion (Lima-Mendez et al., 2015).

## Summary and perspectives

Life on earth was exclusively microbial for most of its history, and is still predominantly so. Microbiologists have been striving to catalog, understand and manage this wealth of life for almost 250 years, and yet been severely limited by technical development. Historically, while general ecology has been based on direct observation combined with mathematical modeling, breakthroughs in microbial ecology have been coupled to technological advance. With recent advances in technologies such as microfluidics and high-throughput DNA sequencing, as well as the steady growth of computational methods and processing capacity, the pace of advance in microbial ecology has been greatly increased. In addition to these approaches, microbiologists can now use metagenomics, metatranscriptomics, metaproteomics, metabolomics, single-cell genome sequencing, genome binning, flow cytometry, cell sorting, high-throughput image analysis and nanoSIMS (nanoscale mass spectrometry), together providing a wide array of complementary techniques for assessing microbial phylogeny and activity in bulk as well as at the single-cell level.

While the work of mapping and modeling microbial life on earth will remain an open field of basic scientific inquiry, it is important to also consider the potential medical and technological applications of these studies. From alternative fuel sources to environmental decontamination, antibiotic resistance to prevention and treatment of immunological and metabolic disorders, many of the biggest challenges of our times may soon find their answers in the myriad of strategies microorganisms adapt to survive, compete, cooperate and thrive on earth. It is therefore crucial that the full potential, as well as the caveats and biases, of established and nascent microbiology approaches are understood.

## Author contributions

LH and AA participated in the study design and the elaboration of the manuscript.

### Conflict of interest statement

The authors declare that the research was conducted in the absence of any commercial or financial relationships that could be construed as a potential conflict of interest.

## Supplementary material

Supplementary File 1 R code used in figures. Figures 3–6, 8 were created in R (v.3.3.1; R Development Core Team, 2016) using packages RGP (v.0.4-1; Flasch et al., 2014), Vegan (v.2.4-3; Oksanen et al., 2017) vioplot (v0.2; Adler, 2005), car (v.2.1-4; Fox and Weisberg, 2011), hexbin (v.1.27.1; Carr et al., 2016), RColorBrewer (v.1.1-2; Neuwirth, 2014), gplots (v.3.0.1; Warnes et al., 2016), and MASS (v7.3-47; Venables and Ripley, 2002). The code used is available in the Supplementary File.
